# The Nordic countries on top of the world – what next?

**DOI:** 10.1080/02813432.2018.1533668

**Published:** 2018-11-05

**Authors:** Päivi E. Korhonen

**Affiliations:** Department of General Practice, University of Turku and Turku University Hospital, Turku, Finland

“Whenever statistics are available, it is folly not to use them.”

British physician and molecular biologist John Cairns

The Global Burden of Disease Study has since 1990 provided reliable measures of disease, suffering and death globally. These gloomy statistics can be turned to advantage by recognizing the major health problems at the global and national levels and finding ways to resolve them.

In June 2018, *The Lancet* published the Global Burden of Disease Study 2016 data on health-care access and quality in 195 countries and territories [1]. The nations were listed in order from best to worst according to the Healthcare Access and Quality (HAQ) Index, which provides an overall score of 0–100 for personal health-care access and quality by location over time. The HAQ Index was constructed using 32 causes of death which should not occur in the presence of effective health care. Each of these causes was also transformed to a scale of 0–100, with a zero score as the worst percentile (1st) observed between 1990 and 2016, and a score of 100 as the best percentile (99th) [1].

The results are flattering to the Nordic countries: Iceland and Norway have the best HAQ Index (97) in the world, Finland is placed 6th (HAQ Index 96), Sweden 8th (95), and Denmark 17th (92). It is worth noting that the UK is placed 23rd (90), the US 29th (89), and Estonia 31st (86). The worst HAQ Index (19) was reported in Somalia and in the Central African Republic. It seems that performance on the HAQ Index is highly correlated with country-level total health spending per capita (*r* = 0.94) and the number of physicians, nurses, and midwives (*r* = 0.79). Countries that scored 90 or higher had successfully treated vaccine-preventable and other infectious diseases, maternal and child health, as well as causes requiring complex case management such as ischemic heart disease, diabetes, chronic kidney disease, etc. [1].

A closer look at the cause-specific metrics reveals that the Nordic countries must still do a lot of work in detecting and treating skin cancers (scores ranging 53–75, while Australia scores 100). Maybe surprisingly, the Nordic countries might also do better in the case of diabetes (scores 78–86, except for Iceland with a full 100).

A score of lower than 90 indicates health areas where progress can be achieved. Thus, in addition to skin cancer and diabetes, the Nordic country-level worklists might be as follows (scores under 80 in parentheses).

Iceland: lower respiratory infections (score 76), ischemic heart disease, epilepsy.

Norway: peptic ulcer, epilepsy (score 78), congenital heart disease.

Finland: ischemic heart disease (score 78), stroke, hypertensive heart disease (score 77), peptic ulcer, epilepsy, congenital heart disease.

Sweden: lower respiratory infections, cervical cancer, colon cancer, leukaemia (score 79), ischemic heart disease, peptic ulcer.

Denmark: lower respiratory infections, neonatal disorders (79), cancers (breast, cervical, colon, lymphoma), stroke, peptic ulcer (75), epilepsy, chronic kidney disease, congenital heart disease [1].

This information raises many questions. Why is the score for access to or quality of lower respiratory infections lower in Iceland (76) than in the other Nordic countries (84–100)? Why does Denmark record a particularly low score (78) on diabetes although the Danish diabetes research is world famous? Why is the score of 79 for leukaemia in Sweden or the score of 78 for epilepsy in Norway lower than in the other Nordic countries (90–99 and 84–90, respectively)? Why is hypertension management so difficult in Finland? Could Finns learn about it from Norwegians and Danes who score 100? Perhaps overworked general practitioners could delegate some tasks to nurse practitioners [2]? Could we all work together with Estonians who score only 26 for hypertension, although the country has made tremendous progress in health care after regaining independence in 1991? The HAQ Index of Estonia has indeed risen from 68 to 86 during the years 1990–2016. At the same time, the Nordic countries also have increased their HAQ Index scores, albeit from a much higher basis: Denmark 81–92, Finland 81–96, Iceland 87–97, Norway 84–97, and Sweden 85–95 [1].

It is clear that the HAQ Index cannot account for all factors related to health-care access and quality, nor does it distinguish between the effects of primary and secondary care [3]. If accessibility and quality of health care are not separately analyzed, differences in these might cause difficulties in interpretation of the results. Given that the major challenges in improving health-care access and quality of care in the Nordic countries concern non-communicable disease management (especially diabetes, cardiovascular disease, and cancer), the Global Burden of Disease statistics are aimed not only at health-care professionals but also at politicians and policy makers. Hopefully they also realize that the current health-care systems in all of the Nordic countries are the best in the world, and that their bedrock, primary care, should not be torn down in the new health-care reforms going on in Norway [4] and in Finland [5]. The past success and current challenges in health care unite the Nordic countries and call for collaboration to learn best practices from each other.
